# Epidemiology and risk factors of *Escherichia coli* bloodstream infections associated with extended-spectrum beta-lactamase production: a national surveillance and data linkage study, Finland, 2018 to 2023

**DOI:** 10.2807/1560-7917.ES.2025.30.40.2500196

**Published:** 2025-10-09

**Authors:** Heikki Ilmavirta, Jukka Ollgren, Kati Räisänen, Tuure Kinnunen, Jari Jalava, Outi Lyytikäinen

**Affiliations:** 1 Department of Clinical Microbiology, Institute of Clinical Medicine, University of Eastern Finland, Kuopio, Finland; 2 ISLAB Laboratory Centre, Kuopio, Finland; 3 Department of Public Health, Finnish Institute for Health and Welfare (THL), Helsinki, Finland

**Keywords:** Escherichia coli, Bloodstream infection, Bacteraemia, Extended-spectrum beta-lactamase, Drug resistance bacterial, Epidemiology, Incidence, Case fatality

## Abstract

**BACKGROUND:**

*Escherichia coli* (EC) is the leading cause of bloodstream infections (BSI). The emergence of extended-spectrum beta-lactamase (ESBL) production in EC is concerning, as it may worsen infection outcomes.

**AIM:**

We aimed to assess the incidence and outcome of ESBL-EC and non-ESBL-EC BSIs in Finland in 2018–2023 and identify factors associated with death.

**METHODS:**

Data from national registers were used to identify EC BSIs and to determine infection origin, patient comorbidities and death within 30 days. Isolates resistant or susceptible with increased exposure to third-generation cephalosporins were defined as ESBL-producing. Trends were analysed using a binomial regression model with log link. Factors associated with 30-day case-fatality were evaluated using a multivariable logistic regression model.

**RESULTS:**

In total, 33,586 EC BSIs were identified, of which 1,916 (5.7%) were ESBL-EC BSIs. The annual incidence of ESBL-EC BSIs decreased from 7.2/100,000 to 4.9/100,000, being 3.3-fold larger for healthcare-associated than community-acquired ESBL-EC BSIs. Non-ESBL-EC BSIs showed similar but weaker trends. The 30-day case-fatality rate was 1.3-fold higher for ESBL-EC than non-ESBL-EC BSIs and 1.7–3.2-fold higher for healthcare-associated than community-acquired BSIs. Factors associated with 30-day case-fatality included age, comorbidity, male sex, and healthcare association and ESBL in patients with no or less severe comorbidities.

**CONCLUSION:**

We observed a decline in EC BSIs in Finland in 2018-2023, especially those caused by ESBL-EC and healthcare-associated BSIs. ESBL-EC BSIs were associated with 30-day case-fatality only among patients with low comorbidity, a phenomenon requiring further investigation. Continuous surveillance of BSI pathogens, also covering BSI outcome, is essential.

**Key public health message**

**What did you want to address in this study and why?**

*Escherichia coli* is the leading cause of bloodstream infections, and emergence of antimicrobial resistance complicates the treatment and may increase the risk of death. We studied factors associated with death in *E. coli* bloodstream infections in Finland 2018–2023, which covered the COVID-19 pandemic years, with particular interest in extended-spectrum beta-lactamase (ESBL) production, an important type of antimicrobial resistance.

**What have we learnt from this study?**

During the study period, the number of new bloodstream infections caused by ESBL-producing *E. coli* decreased more in healthcare settings than in the community. Increasing age, other diseases, male sex and infection acquired in a healthcare setting were associated with higher risk of death, but ESBL-production only in patients with no or less severe other diseases.

**What are the implications of your findings for public health?**

The decline in the number of new bloodstream infections of ESBL-producing *E. coli* may reflect multiple factors associated with the COVID-19 pandemic and related infection control measures and, thus, be temporary. Monitoring infection outcomes can provide valuable insights when assessing the impact of antimicrobial resistance, infection origin and other factors on BSIs. Our results may be useful when evaluating treatment and prevention guidelines for *E. coli*.

## Introduction

Bloodstream infections (BSI), often caused by *Escherichia coli*, lead to considerable and increasing morbidity and mortality both in Finland [1,2] and worldwide [3,4]. Antimicrobial resistance (AMR) complicates the treatment of *E. coli* BSIs, leading to a higher mortality risk [4]. Hence, the emergence of extended-spectrum beta-lactamase (ESBL) production, the major cause of resistance to third-generation cephalosporins (3GC) among *E. coli* [5], is concerning.

Previous systematic reviews demonstrate increased risk of mortality due to infections caused by ESBL-producing Enterobacterales compared with non-ESBL-producing [6]. However, most previous studies have been based on rather small patient cohorts. Larger, population-based studies are needed to better understand the burden and risk factors of BSIs in different regions and populations, and to establish more effective preventive interventions [7]. A few population-based studies conducted in recent years have included outcome data for *E. coli* BSIs. However, their results vary in whether ESBL was identified as a risk factor for case fatality or not. In one study conducted 2014–2018, ESBL was significantly associated with a higher 30-day case-fatality risk [4], whereas this was not observed in two other recent studies [8,9].

In the context of ESBL-producing *E. coli* (ESBL-EC), previous population-based studies in Finland demonstrated that from 2008 to 2019, the proportion of ESBL-EC increased annually around 9% among *E. coli* BSIs [10], but this increasing trend reversed rapidly after the onset of the COVID-19 pandemic in 2020 [11]. However, the effect of this reversed trend on the outcome of ESBL-EC BSIs, as well as whether these changes were similar in the community and healthcare settings, are unknown.

The current study aimed to evaluate the incidence and outcome of ESBL-EC and non-ESBL-EC BSIs in Finland 2018–2023 by using laboratory-based surveillance data linked to other national registers. In addition, we explored factors associated with 2, 7 and 30-day case-fatality of these infections to identify potential targets for preventive interventions.

## Methods

### Study setting and population

The healthcare system in Finland (population: 5.5 million in 2018 and 5.6 million in 2023 [12]) is organised into 20 mainland healthcare districts and one on the islands of Åland. The National Infectious Disease Register (NIDR) is maintained by the Finnish Institute for Health and Welfare [13]. For blood cultures, all clinical microbiology laboratories notify the NIDR electronically of the detection of bacteria and fungi [14]. These notifications include specimen date, type of microbe, data on reduced susceptibility or resistance to certain antimicrobials, and the date of birth, sex, place of residence and national identity code of the patient. To form a single case for each BSI episode, multiple notifications of the same microbe with the same national identity code occurring within 3 months of each other are merged by NIDR. The antimicrobial susceptibility testing and interpretation of the isolates were performed in the clinical microbiology laboratories according to the European Committee on Antimicrobial Susceptibility Testing (EUCAST) guidelines [15]. For *E. coli*, the susceptibility data to 3GCs and carbapenems are sent to the NIDR.

In this retrospective study, we used NIDR data to identify all BSI episodes caused by *E. coli* in Finland from 2018 to 2023. We included all *E. coli* BSI episodes with valid national identity codes, 132 BSI episodes with invalid national identity codes were excluded. We used the Population Information System (PIS) [16] to retrieve the date of death by linking database information with the patient’s national identity code. To obtain information on patient hospitalisation, including origin of the infection (community or healthcare setting), and current and prior (1 year) diagnosis codes, we linked the patient’s national identity codes to the National Hospital Discharge Register (HILMO).

### Definitions

We defined *E. coli* BSIs as ESBL-EC BSIs when *E. coli* was reported as resistant or intermediately susceptible (or susceptible with increased exposure) to 3GCs, and *E. coli* BSIs without that information were defined as non-ESBL-EC BSIs (Box).

Box **Definition of healthcare-associated and community-acquired bloodstream infections caused by *Escherichia coli*, Finland, 2018–2023**

**Bloodstream infection (BSI):**

• Presence of viable microorganisms in the bloodstream, in this study detection of *Escherichia coli* from a blood culture specimen.

**Healthcare-associated (HA) BSI:**

• Blood culture specimen taken > 2 days after hospitalisation or within 2 days of discharge or patient transferred from another healthcare facility.

**Community-acquired (CA) BSI:**

• No prior hospitalisation within 2 days of admission AND

• Blood culture specimen taken ≤ 2 days after hospitalisation.

We defined *E. coli* BSIs as healthcare-associated (HA) when the first positive blood culture of the BSI episode was collected > 2 days after admission to hospital or within 2 days of discharge [17]. In addition, we defined cases as HA if patients were transferred from another healthcare facility. We defined cases as community-acquired (CA) if the patients had no prior hospitalisation within 2 days of hospital admission and the first positive blood culture specimen was collected ≤ 2 days after hospital admission.

To define the patients’ underlying illnesses, we used a validated algorithm for the Charlson comorbidity index (CCI), which was based on the International Classification of Diseases (ICD), 10th Revision [18,19].

### Analyses and statistics

We calculated case-fatality rates (%): deaths of cases within 2, 7 and 30 days after the collection of the first positive blood culture specimen from a particular patient according to data from PIS.

In a univariate analysis of categorical variables, we used the chi-square test, using Yates’s correction or Fisher's exact test, as appropriate. To test for the differences in distributions between continuous variables, we used the Kruskal-Wallis test and calculated the p value for the adjusted residual [20].

We used population data from Statistics Finland [12] as denominators to calculate incidence rates. We determined average annual incidence rates according to the total number of episodes and population from 2018 to 2023.

To compare observed trends in annual incidence, proportions of HA vs CA BSIs, and 30-day case-fatality rates, we applied a binomial regression model with log link. For average annual increase (AAI) and trends, we calculated 95% compatibility intervals (CI) and p values; p values of < 0.05 were considered statistically significant.

To identify factors associated with a higher risk for 30-day case-fatality, we used a multivariable logistic regression model. Age group, sex, CCI score, healthcare association, ESBL status, year and region were included as explanatory variables. We performed a backward selection of variables using the Akaike information criteria when evaluating both the best selection of variables and interactions for the final model [21]. In addition, we compared the absolute increases in predictive margins of case-fatality at different time points (2, 7 and 30 days) when all the other variables except the variable of interest were kept in their observed original values [22].

Analyses were carried out using R software version 4.3 (https://www.r-project.org) and Stata 18.0 (StataCorp LLC, https://www.stata.com).

## Results

### Characteristics of patients with bloodstream infections of *Escherichia coli*

From 2018 to 2023, 33,586 *E. coli* BSIs were identified among 31,463 individuals, and 5.7% (1,916/33,586) of these were caused by ESBL-EC (Table 1). Patients with ESBL-EC BSIs were more often aged 20–69 years and more frequently male than those with non-ESBL-EC BSIs (p < 0.01). The patients with ESBL-EC BSIs also had higher CCI scores (CCI ≥ 1: 56.8%; 1,020/1,795) than patients with non-ESBL-EC BSIs (CCI ≥ 1: 45.6%; 13,211/28,994), presented in Supplementary Table S1: Comparison of underlying comorbidities between ESBL-EC and non-ESCL-EC BSIs. Moreover, ESBL-EC BSIs were more frequently HA (417/1,916; 21.8%) than non-ESBL-EC BSIs (4,554/31,670; 14.4%) (p < 0.01). Furthermore, case-fatality rates within 2, 7 and 30 days were 1.3–1.4-fold higher among patients with ESBL-EC BSI than among those with non-ESBL-EC BSI.

**Table 1:** Univariate analysis of characteristics of patients with bloodstream infections of extended-spectrum beta-lactamase (ESBL)-producing and non-ESBL-producing *Escherichia coli*, Finland, 2018–2023 (n = 33,586)

Characteristics	*E. coli*, total (n = 33,586)		ESBL-EC (n = 1,916)		non-ESBL-EC (n = 31,670)		p value	p value, adjusted residual
	n	%^a^	n	%^a^	n	%^a^		
Age (years)								
< 1	214	0.6	6	0.3	208	0.7	< 0.01	0.033
1–19	275	0.8	11	0.6	264	0.8		0.110
20–69	10,624	31.6	661	34.5	9,963	31.5		< 0.01
≥ 70	22,473	66.9	1,238	64.6	21,235	67.1		0.014
Median	76		76		76		0.030	
Range	0–103		0–102		0–103			
Sex								
Male	13,955	41.6	975	50.9	12,980	41.0	< 0.01	
Female	19,631	58.4	941	49.1	18,690	59.0		
Comorbidity (CCI score)								
With a score	30,789	91.7	1,795	93.7	28,994	91.6	Not applicable	
0	16,558	53.8	775	43.2	15,783	54.4	< 0.01	< 0.01
1–2	11,301	36.7	775	43.2	10,526	36.3		< 0.01
> 2	2,930	9.5	245	13.6	2,685	9.3		< 0.01
Not available	2,797	8.3	121	6.3	2,676	8.4	Not applicable	
Median	0		1		0		< 0.01	
Range	0–15		0–9		0–15			
Infection origin								
Healthcare-associated	4,971	14.8	417	21.8	4,554	14.4	< 0.01	
Community-acquired	28,615	85.2	1,499	78.2	27,116	85.6		
Case-fatality rate								
2-day	982	2.9	73	3.8	909	2.9	0.018	
7-day	1,709	5.1	129	6.7	1,580	5.0	< 0.01	
30-day	3,256	9.7	246	12.8	3,010	9.5	< 0.01	

CCI: Charlson comorbidity index; ESBL-EC: extended-spectrum beta-lactamase-producing *Escherichia coli*.

^a^ Percentages refer to the total number of known cases in each column.

### Annual incidence of bloodstream infections of *Escherichia coli*

**Figure 1:**
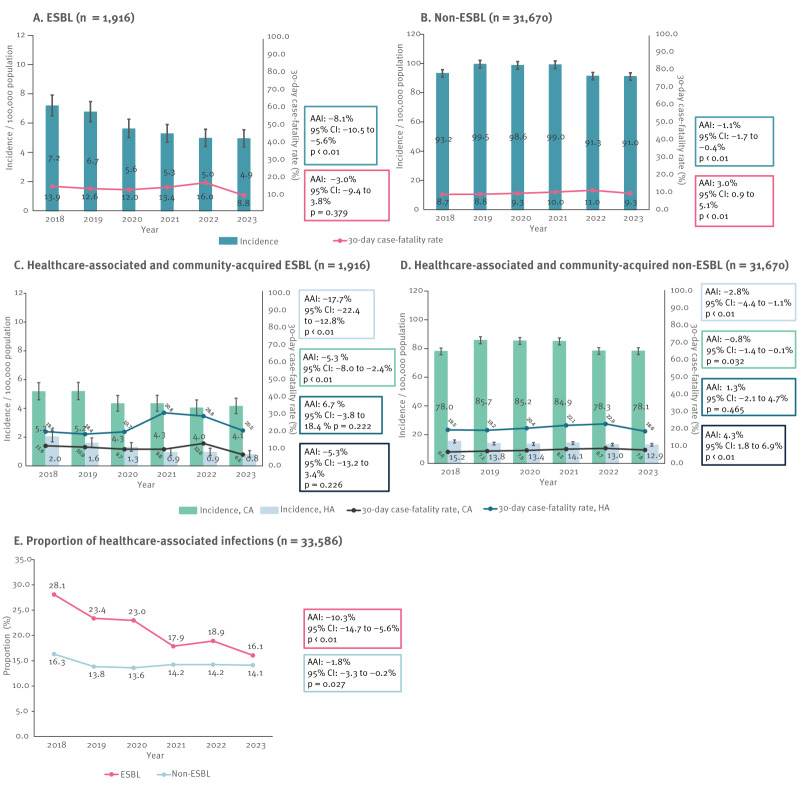
Annual incidence (cases/100,000 population), 30-day case-fatality rate, origin and proportion of bloodstream infections caused by extended-spectrum beta-lactamase (ESBL)-producing and non-ESBL-producing *Escherichia coli*, Finland, 2018–2023 (n = 33,586)

AAI: average annual increase; BSI: bloodstream infection; CA: community-acquired; CI: compatibility interval; ESBL: extended-spectrum beta-lactamase-producing *Escherichia coli*; HA: healthcare-associated.

From 2018 to 2023, a significant decreasing trend in the annual incidence of ESBL-EC BSIs, and to a lesser extent also of non-ESBL-EC BSIs, was observed. For ESBL-EC BSIs, the decrease was from 7.2 to 4.9/100,000 population (AAI: −8.1%; 95% CI: −10.5 to −5.6%; p < 0.01) (Figure 1A) and for non-ESBL-EC BSIs, from 93.2 to 91.0/100,000 population (AAI: −1.1%; 95% CI: −1.7 to −0.4%; p < 0.01) (Figure 1B). Timing of the decreases differed, since for ESBL-EC, the decrease occurred in 2019–2023 and for non-ESBL-EC only in 2022–2023. When these results were further stratified based on infection origin (HA or CA), in ESBL-EC, the decrease was 3.3 times larger for HA-BSIs (AAI: −17.7%; 95% CI: −22.4 to −12.8%; p < 0.01) than for CA-BSIs (AAI: −5.3%; 95% CI: −8.0 to −2.4%; p < 0.01) (Figure 1C). A similar phenomenon was also observed for non-ESBL-EC BSIs that were HA (AAI: −2.8%; 95% CI: −4.4 to −1.1%; p < 0.01) or CA (AAI: −0.8%; 95% CI: −1.4 to −0.1%; p = 0.032) (Figure 1D). Consequently, the proportion of HA-BSIs significantly decreased for both ESBL-EC and non-ESBL-EC, but this decrease was 5.7 times larger among HA-BSIs of ESBL-EC (AAI: −10.3%; 95% CI: −14.7 to −5.6%; p < 0.01) compared with HA-BSIs of non-ESBL-EC (AAI: −1.8%; 95% CI: −3.3 to −0.2%; p = 0.027) (Figure 1E).

### Case-fatality rates in bloodstream infections of *Escherichia coli*

The 30-day case-fatality rate of ESBL-EC BSIs varied from 8.8 to 16.0% from 2018 to 2023, being highest in 2022 and lowest in 2023, but no significant trend was observed during the study period (AAI: −3.0%; 95% CI: −9.4 to 3.8%; p = 0.379) (Figure 1A). For non-ESBL-EC BSIs, the 30-day case-fatality rate varied from 8.7% in 2018 to 11.0% in 2022 with a slightly increasing trend (AAI: 3.0%; 95% CI: 0.9 to 5.1%; p < 0.01) (Figure 1B). When the results were further stratified by origin of infection (HA or CA), the case-fatality rate was consistently higher for HA-BSIs than CA-BSIs for both ESBL-EC and non-ESBL-EC (1.7–3.2-fold higher for HA-BSI of ESBL-EC and 2.4–3.0-fold higher for non-ESBL-EC) (Figure 1C and D). Of note, among HA-BSIs of ESBL-EC, the 30-day case-fatality rate appeared somewhat higher in 2021–2022 (28.8–30.8%), a period coinciding with the COVID-19 pandemic of 11 March 2020–5 May 2023, compared with the pre-pandemic years 2018–2019 (18.4–19.8%) and also the year 2023 (20.5%) (Figure 1C).

### Risk factors for death in bloodstream infections of *Escherichia coli*

**Figure 2:**
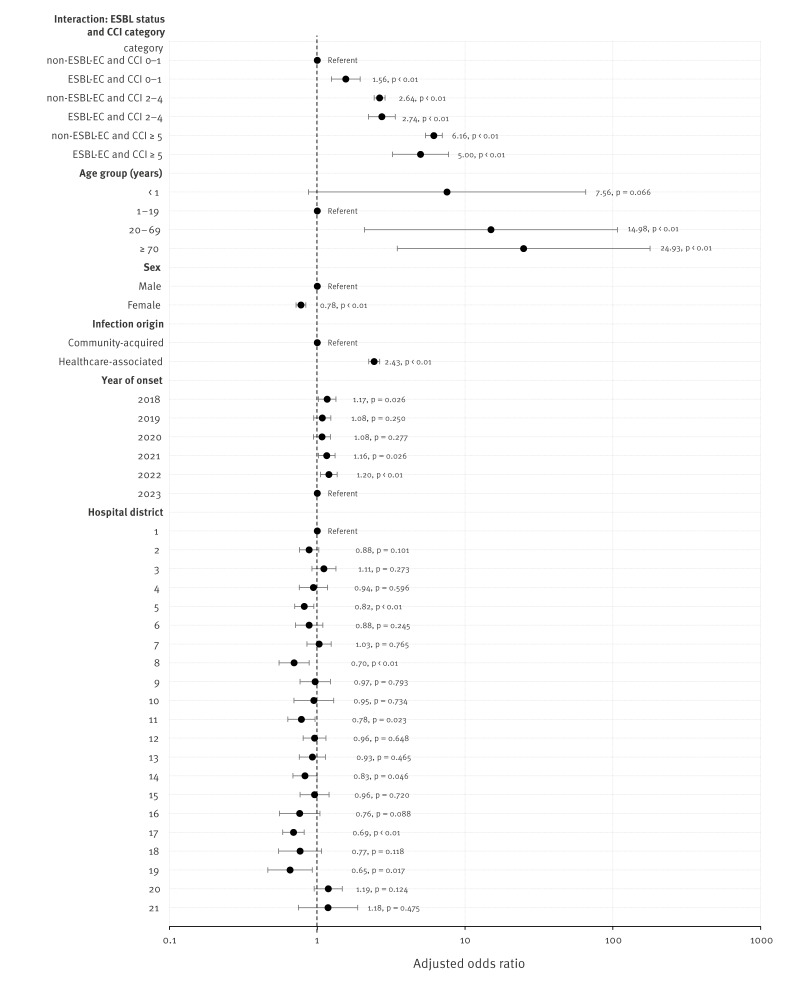
Adjusted odds ratios for 30-day case-fatality of *Escherichia coli* bloodstream infections, by patient characteristics, Finland, 2018–2023 (n = 33,586)

CCI: Charlson comorbidity index; ESBL-EC: extended-spectrum beta-lactamase-producing *Escherichia coli*.

Using a multivariable logistic regression model, a higher CCI score (CCI ≥ 2) was associated with a higher adjusted OR (aOR) for 30-day case-fatality for *E. coli* BSIs (Figure 2). Interestingly, a low CCI score of 0–1 and ESBL-EC was associated with a higher risk for death within 30 days compared with non-ESBL-EC BSIs (aOR = 1.56; p < 0.01). The same phenomenon was not, however, observed among patients with higher CCI scores (CCI 2–4 and CCI ≥ 5). In addition, age (≥ 20 years), male sex and HA were associated with a higher risk for death within 30 days. The aORs were also slightly higher for BSIs occurring in 2018, 2021 and 2022 compared with 2023, and there was variation in aORs between healthcare districts; 6 of 21 districts had slightly lower odds for 30-day case-fatality.

Finally, when all other explanatory variables except ESBL status were kept in their observed original values, ESBL alone led to a 1.2-fold higher predictive margin for 30-day case-fatality (11.3% for ESBL-EC compared with 9.6% for non-ESBL-EC BSIs; p = 0.017) during the study period (Table 2). Similar but smaller increases were also observed for case-fatality rates within 2 and 7 days, but these were not statistically significant.

**Table 2:** Predictive margins and absolute increase in case-fatality within 2, 7 and 30 days for bloodstream infections of extended-spectrum beta-lactamase (ESBL)-producing and non-ESBL-producing *Escherichia coli*, Finland, 2018–2023 (n = 33,586)

Variables	2 days		7 days		30 days	
	non-ESBL-EC	ESBL-EC	non-ESBL-EC	ESBL-EC	non-ESBL-EC	ESBL-EC
Predictive margin (%)	2.9	3.2	5.0	5.9	9.6	11.3
95% CI	2.7 to 3.1	2.5 to 4.0	4.8 to 5.3	4.9 to 6.9	9.3 to 9.9	10.0 to 12.7
Absolute increase (%)	0.3		0.9		1.7	
95% CI	−0.4 to 1.1		−0.2 to 1.9		0.3 to 3.1	
p value	0.413		0.100		0.017	

CI: compatibility interval; ESBL-EC: extended-spectrum beta-lactamase-producing *Escherichia coli*.

## Discussion

Our population-based retrospective cohort study demonstrated that ESBLs in *E. coli* BSIs increased the predictive margin for 30-day case-fatality by around 1.2-fold in 2018–2023 in Finland. Moreover, in the multivariable analysis, after accounting for interactions between ESBL-status and the CCI category of the patient, ESBLs were associated with increased risk of death within 30 days of patients with no or less severe comorbidities. Our study also established that the incidence of *E. coli* BSIs decreased within the Finnish population during the study period, which coincided with the COVID-19 pandemic. This decrease was larger for ESBL-EC BSIs and for those occurring in the healthcare settings. The 30-day case-fatality rate, however, remained higher for ESBL-EC compared with non-ESBL-EC BSIs, and also for *E. coli* BSIs occurring in the healthcare compared with community settings.

During the study period, we noted a 30-day case-fatality rate of 9.7% for *E. coli* BSIs. This strongly concurs with figures from recent population-based studies: 9.6% (range: 7.2–12.8%) in a multinational 2014–2018 study from Finland, Sweden and Canada [4], 10.1% in a study from Queensland, Australia 2000–2019 [9], and 11.5% in a study from British Columbia, Canada 2010–2020 [8]. For ESBL-EC BSIs, our study demonstrated a ca 1.3-fold higher 30-day case-fatality rate compared with non-ESBL-EC BSIs (12.8% vs 9.5%), which is similar to the figures reported in the previous study from Queensland, Australia (12.8% vs 10.0%) [9].

To analyse the association between ESBLs and 30-day case-fatality using a multivariable regression model, we used an interaction between ESBL-status and comorbidities identified by the Akaike information criteria. Here, we demonstrated that ESBLs were associated with increased risk of death within 30 days of patients with no or less severe comorbidities (CCI score: 0–1). Among patients with more severe comorbidities (CCI score ≥ 2), the risk of death from *E. coli* BSIs was high regardless of ESBL-status (2.6–6.2 times higher than for CCI group 0–1) and ESBLs were not associated with increased risk of death within 30 days. However, we cannot exclude that this was related to competing risk factors, which we could not account for, and therefore, the interpretation should be taken with caution in these already high-risk patients. Alternatively, the current empirical treatment guidelines and diagnostics of BSIs may already cover ESBL-EC efficiently in patients with high CCI scores. To evaluate the impact of ESBLs in *E. coli* BSIs on the risk of death at the population level, we calculated predictive margins for 30-day case-fatality in ESBL-EC BSIs and non-ESBL-EC BSIs. This analysis showed around 1.2-fold absolute increase (1.7%; range: 0.3–3.1%) in the predictive margin for 30-day case-fatality due to ESBLs. This indicates that, potentially, 0.3–3.1% (1/333–1/32) of deaths related to EC BSIs may have been attributable to ESBL-EC BSIs from 2018 to 2023 in Finland. However, the observed range is wide, and the finding can unlikely be generalised to other contexts. The results of previous studies vary in whether ESBLs or 3GC resistance have been reported as independent risk factors for 30-day case-fatality. In the multinational study from Finland, Sweden and Canada, 3GC resistance was associated with 30-day case-fatality risk [4]. However, the study material did not include comorbidity data, and only interaction of age and sex was used in the multivariable regression model. In contrast, in the study from British Columbia, Canada, ESBLs in *E. coli* BSIs were only considered a risk factor for longer hospital post-infection length of stay (LOS) in patients surviving the infection but not associated with higher 30-day case-fatality [8]. That study included comorbidity data, but it did not use interactions in the model. Of note, the study had detailed clinical data and covered a period of 10 years, but the total number of ESBL-EC BSIs (n = 91) was rather small, limiting statistical power. Similarly, in a Danish population-based cohort study 2007–2017, ESBL-EC BSI was associated with increased LOS, but not with increased 30-day or 1-year mortality for community-onset *E. coli* bacteraemia, although the 30-day mortality appeared higher for ESBL-EC BSIs compared with non-ESBL-EC BSIs [23]. However, this study included only community-onset infections. A similar phenomenon was also reported in the study from Queensland, Australia, in which ESBLs were again associated with longer hospital LOS and risk of recurrence of *E. coli* BSIs [9]. However, it did not significantly increase case-fatality risk, although case fatality rate tended to be higher in ESBL-EC BSIs than in non-ESBL-EC BSIs.

In addition, in our multivariable regression model, increasing age, HA and male sex were associated with a higher risk of death within 30 days among patients with *E. coli* BSIs. Age and sex, as well as comorbidities, are all non-modifiable risk factors and, therefore, not amenable to patient-level interventions unlike HA. These findings are mostly in line with recent previous studies, in which older age and HA-BSIs were consistently associated with a higher 30-day case-fatality risk [4,8,9], along with increasing CCI score [8,9] and male sex [4,9].

In the current study, the incidence of ESBL-EC BSIs decreased rapidly (AAI: −8.1%) during the study period starting in 2019 and covering the COVID-19 pandemic period of 2020–2023. Among non-ESBL-EC BSIs, this decrease was observed at a much lower extent (AAI: −1.1%) and only started in 2022. The observed decrease in the incidence of ESBL-EC BSIs is in line with our previous Finnish population-based study, which used different national registry data from years 2018–2022 [11]. Similar decreasing trends in the incidence and/or the proportion of ESBL or 3GC-resistant *E. coli* BSIs have also been observed in a retrospective ecological analysis from South West England in 2019–2021 [24]. Societal restrictions, such as less social mixing, reduced travel, alterations in patient population and improved hand hygiene during the COVID-19 pandemic years, all reducing the transmission of *E. coli,* have been suggested as potential explanatory factors for the phenomenon [11,24]. Our current study showed that the decrease in the incidence of ESBL-EC BSIs happened simultaneously both within the community and healthcare settings, but the decrease was around threefold larger for HA infections. It is possible that restriction-related infection prevention and control (IPC) measures were implemented more efficiently in the healthcare settings compared with communities. In the community settings, a major contributor for the decrease may have been strongly decreased international travel during the pandemic years starting in 2020 in Finland [25,26], as this likely heavily influenced the acquisition and cross-border import of ESBL-EC by Finnish travellers [27,28]. Also, the concurrent decrease of 14.9% in antimicrobial consumption in Finland in 2019–2022 observed in the European Surveillance of Antimicrobial Consumption Network (ESAC-Net) report for 2022 [29] may have contributed to the observed decrease in ESBL-EC BSIs. Finland was among four other European Union/European Economic Area (EU/EAA) countries in which a continuous decrease in the community antimicrobial consumption was observed between 2020 and 2021 in addition to 2019–2020, but in 2022, the consumption increased again [30]. However, the decline in antimicrobial consumption was most evident for antimicrobials prescribed for respiratory tract infections, particularly in children and adolescents, such as azithromycin, amoxicillin and doxycycline [31]. No change in antimicrobial consumption was observed in antimicrobial prescriptions used to treat urinary tract infections, the main cause of ESBL-EC BSIs. Overall, the 30-day case-fatality risk of *E. coli* BSIs appeared to peak during the pandemic years 2021 and 2022, especially for HA-ESBL-EC BSIs. This phenomenon may reflect changes in diagnostic activity, patient population at risk, or predisposing factors for invasive infections during the pandemic years. However, these peaks in HA-ESBL-EC BSIs were based on small numbers and the findings were not statistically significant. Moreover, they appeared to occur concurrently with the high number of COVID-19 deaths in the older adults [32].

Our study has several limitations. Firstly, our definition of ESBL-status (susceptible with increased exposure or resistant to 3GCs) covers also other resistance mechanisms for 3GCs in addition to ESBL, such as hyperproduction of ampicillinase C (AmpC). However, according to the Finnish surveillance report of antimicrobial resistance for 2023, the vast majority (97%) of 3GC-resistant EC blood culture isolates were confirmed ESBL-producers, as the proportions of 3GC-resistant isolates and ESBL-producing isolates were highly similar (6.3% vs 6.1%, respectively) [33]. Secondly, we did not have detailed clinical data for the BSIs, such as the infection focus leading to the BSI, the role of BSI in the chain of events leading to death or the main cause of death. Consequently, every death occurring after a positive *E. coli* blood culture may not have been directly caused by the *E. coli* infection. Therefore, we only studied the association of variables to all-cause 30-day case fatality. This is, however, a common limitation for most population-based studies in which case fatality or mortality is calculated from death for any reason occurring in 30 days after a positive blood culture [34]. Thirdly, we did not have information on prior and initiated antimicrobial therapy and the delay and appropriateness of the treatment, although discordant antimicrobial therapy was independently associated with increased odds of mortality in hospitals in the United States in 2005–2014 [35]. In addition, we had no data on the socioeconomic status, although socioeconomic deprivation has been associated with a higher risk of acquisition of BSIs including *E. coli* [36]. Fourthly, the calculated CCI was limited to certain comorbidities identified in hospitalised patients and, therefore, may not cover all comorbidities, particularly those only diagnosed in outpatient clinics. Fifthly, we did not have information whether the patients were suffering from polymicrobial infections, which have previously been recognised as a major contributor for the 30-day case-fatality risk in *E. coli* BSIs [9]. Sixth, the annual number of deaths among patients with ESBL-EC BSI was rather small and therefore were not further analysed by age group and sex. Finally, the study period covers the period of COVID-19 pandemic, which may involve additional confounding factors that we could not account for. However, we included year and region as explanatory variables in our multivariable analysis and cannot exclude that the pandemic years (2020–2023) and regions may have had a slight impact on the 30-day case-fatality risk calculations.

## Conclusion

Our population-based study demonstrated that increasing age, number of comorbidities, male sex and origin of the infection in the healthcare setting were associated with a higher risk for 30-day case-fatality among patients with *E. coli* BSI. Extended-spectrum beta-lactamases appeared to be associated with increased risk of death in patients with no or less severe comorbidities. In patients with more severe comorbidities, this association was not observed, but this interpretation should be taken with caution due to the possible competing risk of death due to comorbidities as a confounder. The changes in the incidence and case fatality of *E. coli* BSIs in 2018–2023 may have been affected by the COVID-19 pandemic and associated infection control measures and are therefore conceivably temporary. Continuous monitoring of the most common bacteria causing BSIs covering the outcome of the infections is necessary and may provide useful information when evaluating the treatment guidelines.

## Data Availability

Part of the ESBL-EC BSI data are available at the Finnish National Infectious Diseases Register at https://sampo.thl.fi/pivot/prod/en/ttr/cases/fact_ttr_cases. Most of the study data were used under license for the current study and are not publicly available. These data are available from the Finnish Institute for Health and Welfare (THL) on reasonable request.

## Supplementary Data

143000 Supplementary Material
