# Supplementary material for: Epidemiology and risk factors of Escherichia coli bloodstream infections associated with extended-spectrum beta-lactamase production: a national surveillance and data linkage study, Finland, 2018 to 2023
Source: Euro Surveill. 2025 Oct 9;30(40):2500196. doi: 10.2807/1560-7917.ES.2025.30.40.2500196 (PMC12514435; doi:10.2807/1560-7917.ES.2025.30.40.2500196)
Supplement: Supplementary Material [file 25-00196_ILMAVIRTA_Supplement.pdf]

Supplementary material

This supplementary material is hosted by *Eurosurveillance* as supporting information alongside the article *Epidemiology and risk factors of Escherichia coli bloodstream infections associated with extended-spectrum beta-lactamase production: a national surveillance and data linkage study, Finland, 2018 to 2023*, on behalf of the authors, who remain responsible for the accuracy and appropriateness of the content. The same standards for ethics, copyright, attributions and permissions as for the article apply. Supplements are not edited by *Eurosurveillance* and the journal is not responsible for the maintenance of any links or email addresses provided therein.

Supplementary Table S1. A comparison of underlying comorbidities according to Charlson comorbidity index between extended-spectrum beta-lactamase (ESBL)-producing and non-ESBL-producing *E. coli* bloodstream infections, Finland, 2018–2023 (n = 30,789 cases).

Underlying comorbidity	All *E. coli* BSIs (n=30,789)	ESBL-EC BSIs (n=1,795)	non-ESBL-EC BSIs (n=28,994)	p value (Pearson chi2)
	n (%)	n (%)	n (%)	
Myocardial infarction	1,815 (5.9)	121 (6.7)	1,694 (5.8)	0.117
Congestive heart failure	4,121 (13.4)	355 (19.8)	3,766 (13.0)	<0.01
Peripheral vascular disease	1,387 (4.5)	133 (7.4)	1,254 (4.3)	<0.01
Cerebrovascular disease	2,857 (9.3)	215 (12.0)	2,642 (9.1)	<0.01
Dementia	2,447 (8.0)	205 (11.4)	2,242 (7.7)	<0.01
Chronic pulmonary disease	2,173 (7.1)	171 (9.5)	2,002 (6.9)	<0.01
Rheumatic disease	940 (3.1)	80 (4.5)	860 (3.0)	<0.01
Peptic ulcer disease	391 (1.3)	25 (1.4)	366 (1.3)	0.632
Mild liver disease	619 (2.0)	34 (1.9)	585 (2.0)	0.718
Diabetes without chronic complications	3,099 (10.1)	184 (10.3)	2,915 (10.1)	0.788
Diabetes with chronic complications	1,182 (3.84)	133 (7.4)	1,049 (3.6)	<0.01
Hemiplegia or paraplegia	123 (0.4)	13 (0.7)	110 (0.4)	0.025
Renal disease	1,444 (4.7)	140 (7.8)	1,304 (4.5)	<0.01
Malignancy	4,302 (14.0)	279 (15.5)	4,023 (13.9)	0.048
Moderate or severe liver disease	297 (1.0)	16 (0.9)	281 (1.0)	0.743
Metastatic malignancy	1,144 (3.7)	56 (3.1)	1,088 (3.8)	0.169
AIDS/HIV	8 (0.03)	2 (0.1)	6 (0.02)	0.021

BSI, bloodstream infection; ESBL-EC, extended-spectrum beta-lactamase-producing *Escherichia coli.*
